# Beyond lithium: expanding treatment for and management of bipolar disorder

**DOI:** 10.1016/j.ebiom.2024.105350

**Published:** 2024-09-11

**Authors:** eBioMedicine

Bipolar disorder is a mood disorder affecting 1–3% of the global population that substantially contributes to disability and mortality. Characterised by alternating episodes of mania and depression, bipolar disorder involves periods of elevated mood and energy contrasted with phases of low mood. 30–60% of people with bipolar disorder attempt to die by suicide at least once and this rate is currently not decreasing, unlike rates in the general global population. A systematic review and meta-analysis published in October, 2023, in the *Journal of Affective Disorders* found that female individuals with bipolar disorder had more reported attempts to die by suicide than male individuals. Bipolar disorder has a higher recorded prevalence in high-income countries than in low-income and middle-income countries, potentially due to better diagnostics and health-care infrastructure.

There are two main types of bipolar disorder. Bipolar I disorder is characterised by severe manic episodes and might include depressive episodes, whereas bipolar II disorder is characterised by less intense hypomanic episodes and more frequent major depressive episodes. The causes of bipolar disorder are complex, involving interactions between environmental and genetic factors. Early diagnosis and long-term management are crucial but challenging due to heterogeneous presentation of symptoms, cardiovascular comorbidities, and frequent misdiagnosis. Environmental factors are important, influencing both onset and progression. An Article published in *eBioMedicine* in May, 2024, highlighted the role of circadian-rhythm disruptions in people with bipolar disorder, indicating that disturbances in circadian phase preceded mood symptoms and that stabilising these rhythms could improve outcomes.

Lithium has been the gold standard for the treatment of bipolar disorder for seven decades due to its efficacy in preventing mood episodes and reducing risk of dying by suicide. However, until now, its mode of action has been unclear and many individuals have bipolar disorder that is resistant to lithium treatment. Furthermore, substantial health risks, such as nephropathy, can occur if lithium is used for more than 10 years. Research has substantially evolved over the past few decades in appreciating the interplay between genetic factors, environmental factors, and the molecular mechanisms underlying treatment responses. Antipsychotics are now also widely used to treat bipolar disorder symptoms but they are often used concurrently with lithium.

An Article published in June, 2024, in *eBioMedicine* provided information about the mechanism of lithium in neurons and alternative treatment options for people with bipolar disorder. Neurons were derived from induced pluripotent stem cells from people with bipolar disorder. Lithium selectively reversed neuronal hyperactivity in neurons from people with lithium-responsive bipolar disorder through changes in Na^+^ currents and Akt signalling pathways; neurons from people with lithium-non-responsive bipolar disorder had no such changes. These findings indicate the importance of Na^+^ currents and Akt signalling in the mode of action and therapeutic effects of lithium. Akt activators mimicked the effects of lithium, emphasising their potential as alternative treatments in people with lithium-responsive bipolar disorder. Activators of AMP-activated protein kinase (AMPK) similarly reversed the hyperactivity and were more effective in neurons from people with lithium-non-responsive bipolar disorder, suggesting the potential of AMPK-based treatments as an alternative to lithium, particularly in people who do not respond to lithium. These mechanistic insights can inform the development of targeted treatments for people who do and people who don't respond to lithium.

Beyond the potential use of Akt and AMPK activators, a systematic review published in *Frontiers in Psychiatry* in February, 2024, highlighted the therapeutic potential of ketamine and its derivative, esketamine. Results indicated that ketamine was effective and well tolerated for treating depressive symptoms within hours, offering hope for people with treatment-resistant bipolar disorder. Moreover, ketamine has been associated with short-term reductions in suicidality in people with bipolar disorder and major depression. However, ketamine and esketamine remain a third-line intervention due to limitations in evidence quality. Research into non-pharmacological alternatives, such as transcranial magnetic stimulation (TMS), has yielded varying results, with bipolar depression being less effectively treated than unipolar depression. A randomised clinical trial of 24 people with treatment-resistant bipolar disorder, published in July, 2024, in *JAMA Psychiatry*, found that intermittent θ-burst stimulation, a form of repetitive TMS, was associated with significantly reduced depression scores. This finding offers hope to people with treatment-resistant bipolar disorder, particularly in preventing suicidality. However, further trials are needed to ascertain the durability of the treatment and its effectiveness on other symptoms.

An exome-sequencing study published in *Nature Genetics* in April, 2022, identified rare variants in *AKAP11*, a gene interacting with the lithium target GSK3B, implicating it in bipolar disorder and suggesting potential biomarkers for risk of bipolar disorder. *AKAP11* is a common risk gene shared in people with bipolar disorder and schizophrenia. However, the genetic and environmental heterogeneity of bipolar disorder continues to challenge its management. Further clarification surrounding the factors influencing varied responses to lithium and its alternatives is crucial. An April, 2024, study published in *International Journal of Bipolar Disorders* showed that people who responded to lithium were more likely to have bipolar I disorder than people who did not respond to lithium, and that people who did not respond to lithium were more likely to have a history of substance use and interpersonal abuse than people who responded to lithium. Despite some genetic differences associated with the dopaminergic system existing between the types of bipolar, the mechanistic and genetic underpinnings of different bipolar types remain unclear.

Due to the narrow therapeutic index of lithium, artificial-intelligence models and machine-learning approaches are being developed to improve management of bipolar disorder. A cohort genome-wide association study published in *The Lancet Psychiatry* in June, 2022, detailed the use of machine-learning models to predict treatment outcomes, thereby advancing personalised lithium treatment plans for people with bipolar disorder. A *Nature Neuropsychopharmacology* study published in March, 2024, showed that large-language models enhanced with evidence-based guidelines can aid clinical decisions for people with bipolar depression.

In November, 2023, WHO updated treatment recommendations, stating that currently more than 75% of people with mental, neurological, and substance-use disorders do not have access to necessary care. Despite stable age-standardised prevalence from 1990 to 2019, the overall burden of bipolar disorder has increased as non-communicable diseases now contribute more to global health challenges than the declining burden of infectious diseases. Concerningly, the incidence of bipolar disorder among adolescents and young adults aged 10–24 years is increasing globally.

As our knowledge of bipolar disorder advances, future research should continue to develop accessible, well tolerated, effective, and personalised management and treatment strategies, particularly for people with bipolar disorder that is resistant to lithium and its alternatives. *eBioMedicine* encourages submissions that investigate relevant mechanisms and enhance understanding of the causes, management, and treatment of bipolar disorder. Addressing the multifaceted challenges of bipolar disorder requires a collaborative effort to translate diverse scientific discoveries into clinical practice, allowing the burden of bipolar disorder to be reduced and ultimately improving outcomes for people with the disorder.

